# [Retracted] Regulation of p38 MAPK phosphorylation inhibits chondrocyte apoptosis in response to heat stress or mechanical stress

**DOI:** 10.3892/ijmm.2026.5843

**Published:** 2026-05-04

**Authors:** Ken Takebe, Takayuki Nishiyama, Shinya Hayashi, Shingo Hashimoto, Takaaki Fujishiro, Noriyuki Kanzaki, Kohei Kawakita, Kenjiro Iwasa, Ryosuke Kuroda, Masahiro Kurosaka

Int J Mol Med 27: 329-335, 2011; DOI: 10.3892/ijmm.2010.588

Following the publication of the above article, a concerned reader drew the Editor's attention to the fact that, regarding the caspase-9 and cleaved caspase-9 bands in Fig. 5D on p. 333, the bands shown for the '10% 0h' and the '10% 12h' lanes/experiments were strikingly similar, albeit with a small downwards displacement of the cleaved caspase-9 band in the right-hand lane of the gel compared with the left-hand lane. A visual inspection of this figure part also revealed the probable presence of breaks in continuity in the affected gel slices, comparing the left- and the right-hand lanes.

Given that this issue has come to light, the Editor of *International Journal of Molecular Medicine* has decided that this article should be retracted from the publication on the grounds of an overall lack of confidence in these data. The authors were asked for an explanation to account for these concerns, but the Editorial Office did not receive a reply. The Editor sincerely apologizes to the readership for any inconvenience caused, and we thank the reader for drawing this matter to our attention.

